# An overview of bats microbiota and its implication in transmissible diseases

**DOI:** 10.3389/fmicb.2022.1012189

**Published:** 2022-10-20

**Authors:** Luca Federici, Michele Masulli, Vincenzo De Laurenzi, Nerino Allocati

**Affiliations:** ^1^Department of Innovative Technologies in Medicine and Dentistry, University “G. d' Annunzio”, Chieti, Italy; ^2^Center for Advanced Studies and Technology (CAST), University “G. d' Annunzio”, Chieti, Italy

**Keywords:** Chiroptera, bats, wildlife microbiota, Zoonoses, antimicrobial resistance, host–microbe interactions

## Abstract

Recent pandemic events have raised the attention of the public on the interactions between human and environment, with particular regard to the more and more feasible transmission to humans of micro-organisms hosted by wild-type species, due to the increasing interspecies contacts originating from human’s activities. Bats, due to their being flying mammals and their increasing promiscuity with humans, have been recognized as hosts frequently capable of transmitting disease-causing microorganisms. Therefore, it is of considerable interest and importance to have a picture as clear as possible of the microorganisms that are hosted by bats. Here we focus on our current knowledge on bats microbiota. We review the most recent literature on this subject, also in view of the bat’s body compartments, their dietary preferences and their habitat. Several pathogenic bacteria, including many carrying multidrug resistance, are indeed common guests of these small mammals, underlining the importance of preserving their habitat, not only to protect them from anthropogenic activities, but also to minimize the spreading of infectious diseases.

## Introduction

**Figure 1:**
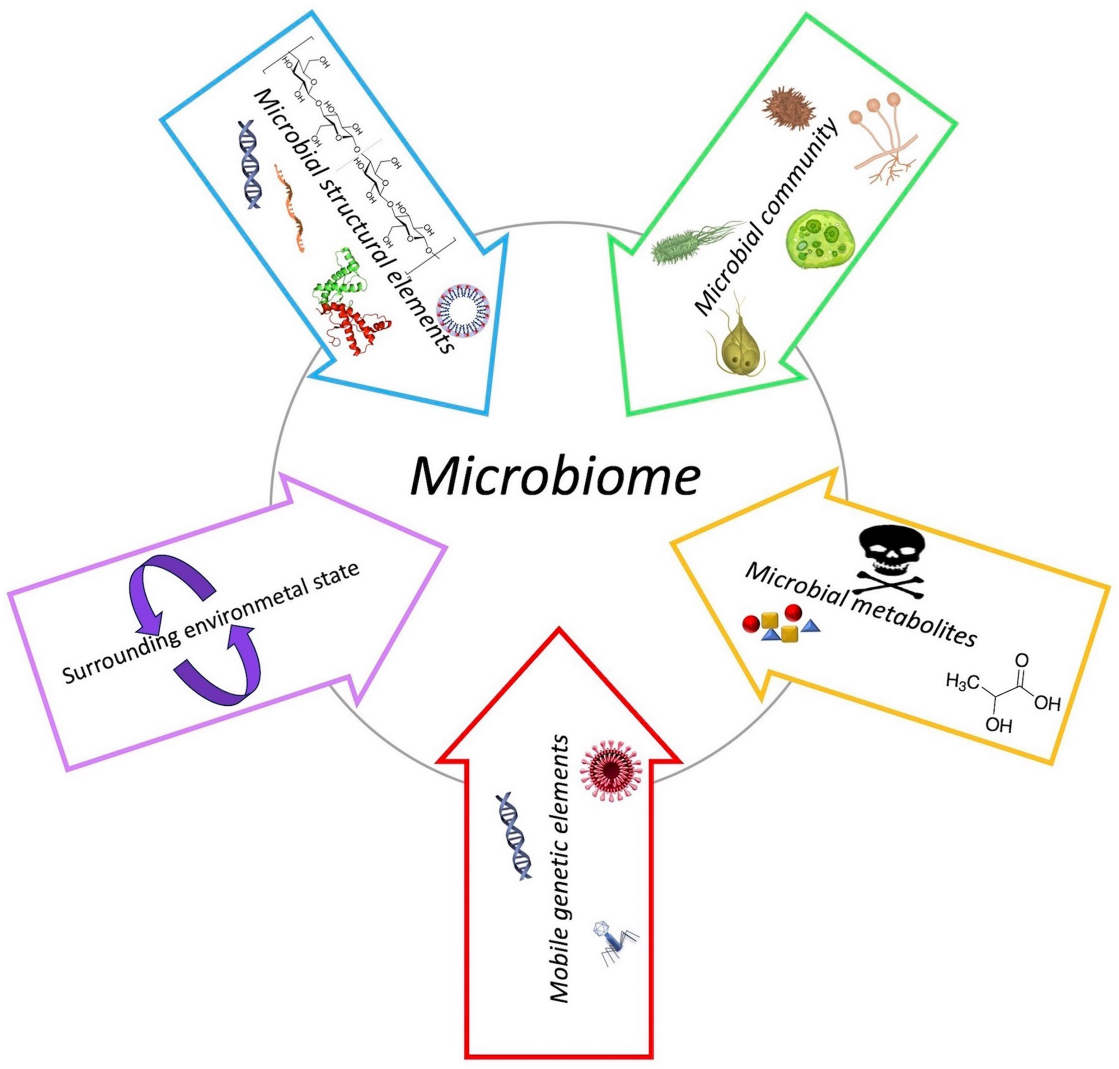
Schematic composition of microbiome.

Recently, the definition of microbiome has been revised enabling a more holistic view of microbial functioning and interaction with its environment (Berg et al., 2020). The microbiome includes the microbiota and their “theatre of activity” represented by microbial structural elements and metabolites, mobile genetic elements – including viruses – and the surrounding environmental conditions (Figure 1; Berg et al., 2020). The microbiota consists of a remarkable heterogeneity and quantity of microorganisms belonging to different prokaryotes and eukaryotes kingdoms which resides inside the body and on the skin of the host to form a complex ecosystem in which bacteria constitute the major part (Berg et al., 2020). In healthy humans, irrespective of age, the internal tissues – such as blood, cerebrospinal fluid, and brain – are usually free of microorganisms. The microorganisms which are constantly present in the other parts of the body, such as on the surface and deep layers of skin, in the saliva and conjunctiva, and in the gastrointestinal tracts, define the normal microbiota. However, under certain circumstances these microorganisms may become pathogenic. The virome is instead composed by all viruses of eukaryotic and prokaryotic cells that are found in or on an organism (Zárate et al., 2017).

Human microbiome research has grown exponentially since the early 2000s, including studies in large populations that have improved our understanding of its diversity and identified potential links with metabolic health and diseases (Fan and Pedersen, 2021). Microbiome has also been studied in other mammals mainly focusing on domestic and charming animals (Ingala et al., 2018a). Conversely, in wildlife species, knowledge about the microbiome remains largely underexplored. Among wildlife species, bats are a good system to take under examination, due to their uniqueness.

Bats are mammals of the order Chiroptera and represent the second largest mammalian order after rodents. They are found almost everywhere in the world with over 1,400 species (Irving et al., 2021). Bats have distinguishing features such as the ability to fly, wide distribution, long-life span and different feeding strategies (Carrillo-Araujo et al., 2015; Irving et al., 2021). These animals are essential members of the global ecosystem and humans benefit from their presence in many ways (Irving et al., 2021). Bats are also well recognized as natural reservoir and carriers of several microorganisms and viruses – some of which cause significant pathogenicity in humans – showing at the same time strong immunity against many of them (Allocati et al., 2016; Hayman, 2016). Several lines of evidence support their role as hosts in the latest emerging zoonotic diseases such as Ebola, MERS, Nipah, and probably also the more recent SARS CoV-type 2 (Zhou et al., 2020), that has killed over 6,300,000 people in the world (source WHO: https://covid19.who.int/). After all, it is known that a common factor of the emerging diseases is the involvement of multiple hosts with the majority of them originating in wildlife (Jones et al., 2008).

Humans are frequently in contact with bats. One of the main causes is the anthropogenic alteration of their natural habitat that forces them to seek alternative sites with consequent interactions with other animals including humans (Daszak et al., 2000). Under this light, full awareness of their microbiota, especially potential pathogens, may be considered essential for public health. Indeed, despite the multiple beneficial roles played by bats in the ecosystem, they are also a reservoir of multi-drug resistant microorganisms, and can contribute to the spreading of resistant bacteria in the environment as well as to transmit them to humans.

Moreover, the precise knowledge of the pathogenic microorganisms forming bat microbiota is important not only for zoonosis control but also for the well-being of these precious allies.

While an increased number of studies have focused on the gut microbiota, little is known about microorganisms hosted by other body sites, which are also potential sources of disease transmission. In this review, we try to provide a comprehensive overview of microbiota diversity in all sites of bats body, highlighting the many differences that may arise from changes in habitat, feeding habits, seasonal changes, coexistence of different bat species and so on. We focus on bacteria, archaea, fungi and protozoa while, as to viruses – many of them are emerging zoonotic viruses – we refer the reader to already published excellent reviews and books (Bats and Viruses: Current Research and Future Trends, 2020; Tan et al., 2021).

## Bacteria

### Skin

The skin is a nonspecific first line of defense against external harm, including pathogenic microorganisms (Grice and Segre, 2011). It is also a composite and dynamic ecosystem, which hosts a complex and variable microbial community, normally harmless. Except for their wing and tail membranes, bats have fur on their entire body. Unlike gut microbial community, the skin microbiota seems to be much more influenced by exposure to the habitat, including environmental microorganisms and abiotic factors such as roosting temperature, spatial proximity and elevation. Recently, it has been observed that the skin microbiota varied over time and among different populations of *Rhinolophus ferrumequinum* (Li A. et al., 2021). Furthermore, differences were found between the microbiota of bats from captive and free-living populations (Winter et al., 2017). Also, the skin microbiota is significantly more abundant and various than gut or oral microbial communities (Lutz et al., 2019). In gut and oral microbiota, Proteobacteria – although with different families in percentage – was the dominant phylum with 60% and 68%, respectively. The skin microbiota was not dominated by one particular bacterial phylum, exhibiting Proteobacteria and Actinobacteria with a percentage of 35% and 23%, respectively (Lutz et al., 2019).

The skin microbiota of two different frugivorous bat species, living in captivity in two different areas were also investigated (Lemieux-Labonté et al., 2016). In the first area, *Artibeus jamaicensis* and *Carollia perspicillata* lived together, while the second environment included only *A. jamaicensis*. In this case, both habitat and bats shaped the composition and diversity of the skin microbiota, with environmental factors having the strongest influence. Indeed, cohabitating *A. jamaicensis* and *C. perspicillata* shared more similar skin microbiota than members of *A. jamaicensis* across the two areas. In this case the predominant phyla were, in decreasing percentage, the following: Actinobacteria, Proteobacteria, Firmicutes, Cyanobacteria, Bacteroidetes and Fusobacteria, with significant differences at order level. The results showed that the skin microbial community of captive bats is shaped both by environment and host species.

The effects of habitat on skin/fur microbiota were also observed by Winter et al. (2017). They analyzed samples from 163 bats collected from wide areas between New Mexico and Arizona, of which 60 were cave-caught and 103 were surface netted. Actinobacteria, Proteobacteria with Alphaproteobacteria and Gammaproteobacteria classes, and Firmicutes phyla made up the most abundant taxa across all bat species. Significant differences in phyla were observed between geographical areas and between cave and surface sampling locations. In particular, Actinobacteria were prevalent in cave-caught bats, whereas Cyanobacteria and Actinobacteria phyla, and in particular the Alphaproteobacteria class, were the most abundant on surface-netted bats (Winter et al., 2017). Moreover, the skin/fur microbiota found on bats caught in caves was more homogeneous than the one found on bats caught on the surface.

The microbiota of 12 different bat species, living in various sites of three North American States (Virginia, New York and Colorado) was studied (Table 1; Avena et al., 2016). The prominent bacterial classes present in both bat and environmental samples were: Gammaproteobacteria, Alphaproteobacteria, Actinobacteria, Betaproteobacteria, Bacilli and Flavobacteria suggesting that these bacterial classes were shared between the host and its environment. Moreover, two common genera – *Pseudomonas* and *Acinetobacter* – found on bats, are typical environmental strains.

**Table 1:** Taxonomic distribution of commonly widespread bacteria in various body sites.

Bat species	Status/Diet	Samples	Detection and identification approach^a^	Tassonomic distribution at level of		Ref.
				Phyla	Genera/Species	
*Skin*						
*Pteropus livingstonii*	Captive/F	Ventral wing	Traditional	Firmicutes	*S. aureus, S. xylosus, S. nepalensis, S. saprophyticus*, *S. simiae*	Fountain et al. (2019)
*Rousettus aegyptiacus*	Captive Free-living/F	Fur	Molecular	Firmicutes Proteobacteria	*Streptococcus salivarius*	Kolodny et al. (2019)
*Myotis* sp.*, Corynorhinus townsendii, Eptesicus fuscus, Antrozous pallidus, Parastrellus hesperus, Lasionycteris noctivagans, Tadarida brasiliensis*	Free-living/I	Entire skin and furred surface	Molecular	Actinobacteria Proteobacteria Firmicutes	—	Winter et al. (2017)
*Artibeus jamaicensis, Carollia perspicillata*	Captive/F	Back and on wing	Molecular	Actinobacteria Proteobacteria Firmicutes Cyanobacteria Bacteroidetes Fusobacteria	—	Lemieux-Labonté et al. (2016)
Eastern U.S.: *Myotis* sp., *Perimyotis subflavus* Colorado: *Myotis* spp., *Eptesicus fuscus*, *Corynorhinus townsendii, Lasiurus cinereus*	Free-living/I	Forearm and muzzle	Molecular	Proteobacteria	*Pseudomonas* sp.*, Acinetobacter* sp.	Avena et al. (2016)
*Leptonycteris yerbabuenae*	Free-living/N	Dorsal patch	Molecular	Firmicutes Proteobacteria Actinoacteria	*Lactococcus, Helcococcus*, *Aggregatibacter*, *Enterococcus, Gallicola*, *Staphylococcus, Clostridium, Anaerococcus*, *Peptostreptococcus*	Gaona et al. (2019a)
*Bacterial strains with biocontrol activity against Pseudogymnoascus destructans*						
*Eptesicus fuscus, Myotis leibii, M. lucifugus, M. sodalis*	Free-living/I	Forearm and muzzle	Traditional Molecular	Proteobacteria	*Pseudomonas fluorescens* group, *Ps. abietaniphila*	Hoyt et al. (2015)
*Rhinolophus ferrumequinum, Myotis petax*	Free-living/I	Wing	Traditional Molecular	Proteobacteria	*Ps. yamanorum, Ps. brenneri, Ps. fragi*	Li Z. et al. (2021)
*Myotis* sp*., Corynorhinus townsendii, Antrozous pallidus, Eptesicus fuscus, Tadarida brasiliensis, Parastrellus hesperus, Lasionycteris noctivagans*	Free-living/I	Wing and tail	Traditional Molecular	Actinobacteria	*Streptomyces* sp., *Rhodococcus* sp., *Streptosporangium* sp., *Luteipulveratus* sp., *Nocardiopsis* sp.	Hamm et al. (2017)
*Eye surface*						
*Desmodus rotundus*	Free-living/S	Cornea and conjunctiva	Traditional	Firmicutes	*Staphylococcus sp., Bacillus* sp*., Micrococcus* sp*., Corynebacterium* sp*., Shigella* sp*., Flavobacterium odoratum*	Leigue Dos Santos et al. (2014)
*Diaemus youngi*	Free-living/S	Cornea and conjunctiva	Traditional	Firmicutes	*Staphylococcus sp., Bacillus* sp*., Streptomyces* sp*., Morganella morganii, Hafnia alvei*	Leigue Dos Santos et al. (2014)
*Artibeus lituratus*	Captive/F	Cornea and conjunctiva	Traditional	Firmicutes	*Staphylococcus* sp*., Bacillus cereus, Corynebacterium* sp.	Leigue Dos Santos et al. (2014)
*Oral cavity*						
*Pteropus livingstonii*	Captive/F	Oropharyngeal mucosae	Traditional	Firmicutes	*S. aureus, S. xylosus, S. nepalensis, S. saprophyticus*, *S. simulans*	Fountain et al. (2019)
*Artibeus* sp*., Dermanura* sp*., Centurio cenex, Sturnira* sp. *Glossophaga morenoi*	Free-living/F Free-living/N FFree-living/N	Oral cavity	Traditional	Proteobacteria	*Bacillus cereus, Xanthomonas* sp.	Galicia et al. (2014)
*Desmodus rotundus*	Free-living/S	Oral cavity	Traditional	Proteobacteria	*Serratia marcescens, S. aureus, S. epidermidis, Aeromonas hydrophila*	Galicia et al. (2014)
*Nycteris thebaica, Miniopterus natalensis, Rhinolophus simulator, Neoromicia capensis*	Free-living/I	Saliva	Molecular	Proteobacteria	*Burkholderia, Helicobacter, Bartonella*	Dietrich et al. (2017)
*Stomach*						
*Rhinolophus luctus*	Free-living/I	Stomach content	Molecular	Firmicutes	*Lactococcus*, *Paeniclostridium*	Sun et al. (2019)
*Murina leucogaster*	Free-living/I	Stomach content	Molecular	Proteobacteria	*Undibacterium*, *Burkholderia*	Sun et al. (2019)
*Cynopterus b. brachyotis*	Free-living/F	Stomach organ	Molecular	Proteobacteria Firmicutes	*Bacillus, Enterobacter, Enterococcus, Klebsiella, Pantoea, Pseudomonas*	Daniel et al. (2013)
*Gut*						
*Frugivores*						
*Rousettus leschenaultia, Cynopterus sphinx*	Free-living	Feces	Molecular	Proteobacteria Firmicutes	*Klebsiella*, *Enterobacter, Weissella*, *Ureaplasma*, *Fructobacillus*	Li et al. (2018)
*Rousettus aegyptiacus*	Free-living	Feces	Molecular	Firmicutes Proteobacteria Actinobacteria	*Direct: Streptococcus*, *Actinobacillus* *Indirect: Streptococcus, Alkalibacterium, Nesterenkonia*	Dietrich and Markotter (2019)
*Rousettus amplexicaudatus*	Free-living	Rectal samples	Molecular	Proteobacteria	*Campylobacter jejuni*	Hatta et al. (2016)
*Nectarivores*						
*Eonycteris spelaea*	Free-living	Fresh feces	Molecular	Proteobacteria Firmicutes	*Klebsiella*, *Enterobacter, Weissella*, *Ureaplasma*, *Fructobacillus*	Li et al. (2018)
*Insectivores*						
*Myotis ricketti, Hipposideros larvatus, Tylonycteris pachypus, Pipistrellus abramus, Scotophilus heathi, Hipposideros armiger*	Free-living	Fresh feces	Molecular	Proteobacteria Firmicutes	*Plesiomonas, Lactobacillus*, *Enterococcus*, *Bacillus*	Li et al. (2018)
*Rhinolophus luctus*	Free-living	Entire intestine content	Molecular	Proteobacteria Firmicutes	*Undibacterium, Paeniclostridium*	Sun et al. (2019)
*Murina leucogaster*	Free-living	Entire intestine content	Molecular	Proteobacteria Firmicutes	*Undibacterium, Enterococcus*	Sun et al. (2019)
*Rhinolophus hipposideros*	Free-living	Feces	Molecular	Proteobacteria	*Anaplasma phagocytophilum*	Afonso and Goydadin (2018)
*Pipistrellus pipistrellus*	Free-living	Intestine	Traditional	Proteobacteria	*Yersinia enterocolitica*	Muhldorfer et al. (2010)
*Miniopterus schreibersii*	Free-living	Small intestine	Traditional	Proteobacteria	*Yersinia enterocolitica*	Imnadze et al. (2020)
*Sanguivores*						
*Desmodus rotundus*	Free-living	Anal cavity	Traditional	Proteobacteria	*Aeromonas hydrophila*	Galicia et al. (2014)
*Kidney*						
*Pteronotus parnellii*	Free-living/I	Kidney organ	Molecular	Spirochaetes	*Leptospira santarosai* *L. borgpetersenii*	Torres-Castro et al. (2020)
*Chiroderma villosum*	Free-living/F	Kidney organ	Molecular	Spirochaetes	*Leptospira noguchii* *L. borgpetersenii*	Torres-Castro et al. (2020)
*Other organs and blood*						
*Desmodus rotundus*	Free-living/S	*Heart, Blood* *Liver*	Traditional Molecular	Proteobacteria	*Bartonella* spp.	Stuckey et al. (2017b) and André et al. (2019)
*Diphylla ecaudata*	Free-living/S	Liver	Molecular	Proteobacteria	*Bartonella* spp.	André et al. (2019)
*Balantiopteryx plicata* *Pteronotus parnellii*	Free-living/I	*Heart, Blood*	Traditional	Proteobacteria	*Bartonella* spp.	Stuckey et al. (2017b)
*Artibeus jamaicensis* *Stumira* spp.	Free-living/P	*Heart, Blood*	Traditional	Proteobacteria	*Bartonella* spp.	Stuckey et al. (2017b)
*Myotis lucifugus*	Free-living/I	Spleen	Molecular	Tenericutes	*Hemotropic mycoplasma* spp.	Mascarelli et al. (2014)
*Miniopterus schreibersii*	Free-living/I	Blood	Molecular	Tenericutes	*Candidatus M. hemohominis*	Millán et al. (2015)
*D. rotundus*	Free-living/S	Blood	Molecular	Tenericutes	*Hemotropic mycoplasma* spp.	Volokhov et al. (2017)
*Pteropus* spp.	Free-living/F	Spleen and brain	Molecular	Tenericutes	*Candidatus M. hemohominis*	Descloux et al. (2021)
*Myotis myotis*	Free-living/I	Liver, lung, spleen	Traditional	Proteobacteria	*Yersinia pseudotuberculosis*	Muhldorfer et al. (2010)
*Carollia perspicillata*	Captive/F	Liver, lung, and spleen	Traditional	Proteobacteria	*Yersinia pseudotuberculosis*	Hahn et al. (2021)
*Pipistrellus pipistrellus*	Free-living/I	Spleen	Traditional	Proteobacteria	*Yersinia enterocolitica*	Muhldorfer et al. (2010)

a Traditional identification methods are based on the isolation in culture media of microorganisms followed by biochemical tests. Molecular techniques rely on the analysis of genomic markers corresponding to nucleic acid sequences directly from the clinical specimen without need for prior culture. When present, both methods are used in succession. Traditional to isolate bacteria of interest and molecular for identification. F, Frugivore; N, Nectarivore; I, Insectivore; S, Sanguivore; P, Phytophage (inclusive of frugivorous and nectarivorous species). The traditional approach allows the study of the sensitivity of microorganism to pharmacological treatment. Molecular biology techniques allow a better identification of the genome present in the samples, but it is difficult to understand if this material belongs to living microorganisms.

In humans, the preponderance of staphylococcal infections is caused by endogenous strains, in particular *Staphylococcus aureus*, that are carried by the infected individual on skin or mucosae. Unlike domestic animals, staphylococcal carriage is poorly documented in wildlife. Several *Staphylococcus* species were found in a captive population at Jersey Zoo (Jersey, Channel Islands, United Kingdom; Fountain et al., 2019). Bats were sampled by swabbing from ventral wing skin and oropharynx, as well as from mouth ejecta and skin lesions. Seventeen coagulase-negative staphylococci, part of normal skin microbiota, were isolated. *Staphylococcus aureus* strain was identified from both healthy and lesioned samples. *Staphylococcus xylosus, Staphylococcus nepalensis, Staphylococcus saprophyticus*, and *S. aureus* were also commonly present, and two species, *S. nepalensis* and *Staphylococcus simiae*, were isolated for the first time on the bat skin (Fountain et al., 2019). Interestingly, in contrast of other wildlife reports, the level of antibiotic resistance was very low suggesting that animals living in areas with limited or absent human activities are less exposed to antibacterial drugs.

Recently, a comparison between fur and gut microbiota was reported, analyzed by using 16S ribosomal RNA (rRNA) gene amplification, of 10 Egyptian fruit bats (*Rousettus aegyptiacus*) in a captive colony and 4 individuals from a wild colony (Kolodny et al., 2019). Both samples showed a high degree of overlap, about 86% of bacterial communities with prevailing Firmicutes (mean 57%) and Proteobacteria (mean 24%) phyla. *Streptococcus salivarius* was the most common species in both groups. In particular, the microbiota composition of wild bats was similar to the captive individuals probably due to the frequent addition of bats – before the sampling period – from the wild to the captive colony or to the similarity of diet (Kolodny et al., 2019). Furthermore, it was found that the fur microbiota changes over time in a manner that is coordinated across the whole colony, probably due to close contact between individuals in the colony. This produces a homogeneous effect in which the fur microbiota of all animals in the colony acts as one. These changes may be influenced by environmental factors – such as diet or climate – as well as endogenous host factors like genetic variability or ecological succession. Similar results were also obtained in others studies (Avena et al., 2016; Lemieux-Labonté et al., 2016; Winter et al., 2017).

The dorsal patch is a odoriferous temporary structure – located in the interscapular region – that the males of pollinating *Leptonycteris yerbabuenae* develop during the mating season (Gaona et al., 2019a). This structure is involved in the attraction of females. The evaluation of the bacterial composition is substantially similar in all interscapular dorsal patch samples of *L. yerbabuenae* males. Most of the shared bacteria belong to genera and families found and described in humans as part of the skin microbiota (*Finegoldia*, *Pasteurellaceae*), associated with wounds or infections (*Helcococcus*, *Enterococcus*) or with the production of fermented products or volatile fatty acids (*Peptostreptococcus*, *Anaerococcus*, *Gallicola*, *Lactococcus*; Table 1). Several chemical compounds were identified exclusively in males with dorsal patch in comparison with males without dorsal patch and females (Muñoz-Romo et al., 2012). It has been suggested that some of these compounds could be the product of bacterial activity and they could affect the behavior of bats playing a key role in the mating strategies of males and females (Muñoz-Romo et al., 2012).

The knowledge of the skin microbiota could prove important in counteracting the white-nose syndrome (WNS), a fungal disease caused by *Pseudogymnoascus destructans* that is responsible for the deaths of millions of bats in North America (see in the Fungi section; Hoyt et al., 2021). Several bat’s skin bacteria are able to inhibit the growth of this fungus such as *Pseudomonas* and *Streptomyces* genera (Hoyt et al., 2015; Hamm et al., 2017; Lemieux-Labonté et al., 2017; Li A. et al., 2021; Li Z. et al., 2021).

Bacteria belonging to the *Pseudomonas* genus – isolated by culture methods and classified by morphotype – were screened to determine their ability to inhibit fungus growth using agar plate challenge assay (Hoyt et al., 2015). Bacteria were sampled using epidermal swabs that were collected by rubbing the forearm and muzzle for each bat. The six bacteria selected were identified by molecular methods and they were used for zone of inhibition assay. All strains were able to significantly inhibit the growth of the fungus (Hoyt et al., 2015). In a more recent paper, three cutaneous bacteria of the *Pseudomonas* genus were isolated and identified from wing membranes (Table 1) that were able to inhibit the growth of the fungus *in vitro* (Li Z. et al., 2021). *Pseudomonas yamanorum*, with the higher inhibition score (about 71%), was selected to analyze the antifungal active molecules produced by the bacterium. A single molecule was identified, i.e., phenazine-1-carboxylic acid, which displayed a minimal inhibitory concentration (MIC) against *P. destructans* of 50.12 μg/ml. Phenazine compounds have been purified by several microorganisms and they are recognized as antimicrobial agents. Furthermore, it was observed that *Ps. yamanorum* produced also a number of volatile organic compounds that significantly inhibited the growth of fungus (Li Z. et al., 2021).

In a recent study, new *Streptomyces* species with antifungal activity were isolated in WNS-free caves located in the regions of New Mexico and Arizona (Hamm et al., 2017). Actinobacteria, and the genus of *Streptomyces*, are ubiquitous and plentiful in caves. These bacteria have anti-fungal activity because of their ability to degrade chitin, a major component of the fungal cell wall, and of targeting ergosterol in the cell membrane of fungi. Thirty-six actinobacteria – of which 32 were *Streptomyces* – were isolated from WNS-free bats (Hamm et al., 2017) and all strains were able to inhibit or stop the growth of the fungus. It is unknown if these bacteria are antagonists of the fungus *in vivo*, but they could serve as natural alternative preventive measures or treatment for bats infected with *P. destructans*. In the future, bacterial strains could be used as biocontrol agents to protect bats exposed to *P. destructans*.

### Eye surface

The eye surface is unique in that it is exposed to the environment but maintains active, both specific and nonspecific, defenses against potentially pathogenic microorganisms of both endogenous and exogenous origin. We found only one study on ocular microbiota in bats (Leigue Dos Santos et al., 2014). Gram-positive bacteria were predominant and coagulase-negative *Staphylococci* were the most frequently isolated from healthy bat eyes. Only four species of Gram-negative bacteria were isolated (Table 1).

### Oral cavity

Differences in pH and buffering capacity in saliva were observed in bats resulting from the different diets (Dumont, 1997). In insectivores, saliva had a significantly higher pH and better buffering capacity than in frugivores. One possible explanation for the higher acidity in the saliva of frugivores could be the protection against potentially harmful microorganisms (Dumont, 1997). The composition and bacterial diversity in the oral and anal regions of frugivorous, nectivorous and hematophagous bat species was characterized in relation to the different diet (Galicia et al., 2014). Among the bacteria that were found, the predominant phylum was Proteobacteria with the family *Enterobacteriaceae*. Statistically significant differences were found between oral and anal samples. Furthermore, different bacterial specificity was observed in nectivores and frugivores in comparison with sanguivores (Table 1). These differences can be explained by the type of diet and/or by the transfer of bacteria from their preys (Galicia et al., 2014). Various *Staphylococcus* species were identified in oropharynx and mouth ejecta in captive fruit bats at Jersey Zoo (Fountain et al., 2019). *Staphylococcus saprophyticus* and *S. aureus* were the dominant species. In saliva samples of four African insectivores, the predominant phylum was Proteobacteria with the endogenous genera from the *Pasteurellaceae* and *Neisseriaceae* families (Dietrich et al., 2017). Various genera that include human opportunistic pathogens such as *Burkholderia* and *Helicobacter* were detected. The potential zoonotic bacterium *Bartonella* was also detected. The presence of *Trypanosoma cruzi* in the saliva of four neotropical bat species in northern Peru was also reported (see in protozoa section).

### Stomach

The secretion of hydrochloric acid in the stomach is primarily meant to denature proteins and activate pepsinogen to initiate the hydrolysis of peptide bonds. However, the acidic pH environment of the human stomach is also considered as a barrier to the colonization by foreign microbes entering the gastrointestinal tract. It is known that the stomach through its acidity acts as an ecological filter influencing the diversity and composition of microorganisms in the vertebrate gut (Beasley et al., 2015). In other animals, the pH of the stomach seems to derive from the type of food they eat (Beasley et al., 2015). For example, species that feed on carrion or similar organisms should require the most restrictive filter through high stomach acidity, as protection from external microorganisms. In herbivores the gastric environment is alkaline, suitable for cellulose digestion and require a least restrictive filter, as the risk of pathogen exposure is lower. In bats, the entire gastrointestinal tract is anatomically simpler than in other mammals, which allows them a short retention time and a reduction of the carried load during the flight (Makanya et al., 2001). Their digestion occurs very quickly and the passage of food through the gastrointestinal tracts was shown to be rapid. The complexity of the morphology is also related to the diet type (see also in gut section; Gadelha-Alves et al., 2008; Strobel et al., 2015). As an example, frugivores – whose food contains large amounts of water, up to 90% – have high digestive and very high absorptive capacities of the intestine due to an extensive microvillous surface area (Makanya et al., 2001). Furthermore, the stomach of fruit bats is particularly rich in parietal cells (80%–90% of the gastric line). The high secretion of hydrochloric acid would allow a rapid digestive process (Tedman and Hall, 1985). Insectivorous bats have developed an enzymatic adaptation to their diet. Chitin is a major component of the exoskeleton cuticle and the peritrophic matrix of the midgut in arthropods. Insectivores can digest chitin providing energy and nutrients. Indeed, digestive chitinolytic activity was detected in the stomach of several insectivorous bat species. It was observed that these bats produced an acidic mammalian chitinase to metabolize chitin with a higher enzymatic activity in the range of pH 5.0–6.0 (Strobel et al., 2013). Digestive chitinolytic activity was also obtained by microorganisms. Several bacterial genera are known to produce chitinase (Whitaker et al., 2004; Veliz et al., 2017). Although these bacteria are not unique to insectivores, to date chitin-producing bacteria have only been found in bats that feed on insects (Whitaker et al., 2004; Veliz et al., 2017; Wang et al., 2022).

In the gastrointestinal tract of two insectivorous bats, *Rhinolophus luctus* and *Murina leucogaster*, a *Citrobacter* strain producing this enzyme was identified (Sun et al., 2019). Furthermore, in their stomach, the dominant genera were *Lactococcus* and *Paeniclostridium* (*Clostridium*) and *Undibacterium* and *Burkholderia*, respectively (Table 1; Sun et al., 2019). In fruit bat *Cynopterus brachyotis brachyotis*, the *Enterobacter* and *Klebsiella* genera – belonging to the *Enterobacteriaceae* family – were the most common, followed by *Bacillus cereus, Pantoea agglomerans* (formerly called *Enterobacter agglomerans*)*, Enterococcus faecalis* and *Pseudomonas aeruginosa* (Table 1; Daniel et al., 2013).

The detection of a relatively small diversity of bacterial species in the stomach as compared to gut (Daniel et al., 2013; Sun et al., 2019) is probably due to the transient microbiota – bacteria are continuously ingested with food and water from diet – which is largely eliminated due to the low pH of the gastric environment (Strobel et al., 2013). An example, *B. cereus* is commonly found in the soil while the presence of *Ps. aeruginosa* in bats is probably due to the contamination of food and water (Daniel et al., 2013).

### Gut

Gut is commonly the most abundant site of microbial colonization. Gut microbiota is a dynamic entity with the composition of the microbial community changing quickly in response to modifications of the host diet and it is closely related to host phylogeny (Carrillo-Araujo et al., 2015). The relationship between hosts and their gut microbiota is considered an evolutionary process of mutual adaptations that is key to biological heterogeneity (Carrillo-Araujo et al., 2015). In human, gut microbiota has an important role in digestion and in regulating the immune response (Fan and Pedersen, 2021), while the role of the gut microbiota in influencing the immune function remains largely unexplored. Like in humans (Rodríguez et al., 2015), in bats gut microbiota changes during the development of the organism mostly due to the effects of dietary and lifestyle changes (Gaona et al., 2019b; Xiao Y. et al., 2019; Edenborough et al., 2020; Yin et al., 2020). Microbiota in gut is composed of bacteria, archaea, protozoa, and fungi. In mammals, the different environments and feeding habits have scarcely affected bacterial phyla of the intestinal microbiota. Although there are over a 100 phyla detected in nature, only a few of these are consistently represented in mammals (Baquero et al., 2021). In bats, the dominant phylum is represented by Proteobacteria followed by – with variable percentages depending on the bat species – Firmicutes, Actinobacteria and Bacteroidetes (in bats relatively poor but instead a dominant phylum in other mammals). As example, in insectivores the range of percentages observed was Proteobacteria (up to 90%), Firmicutes (3%–50%), Actinobacteria (0.01%–19%) and Bacteroides (0.02%–4%; Leon et al., 2018; Vengust et al., 2018; Sun et al., 2019, 2020).

**Figure 2:**
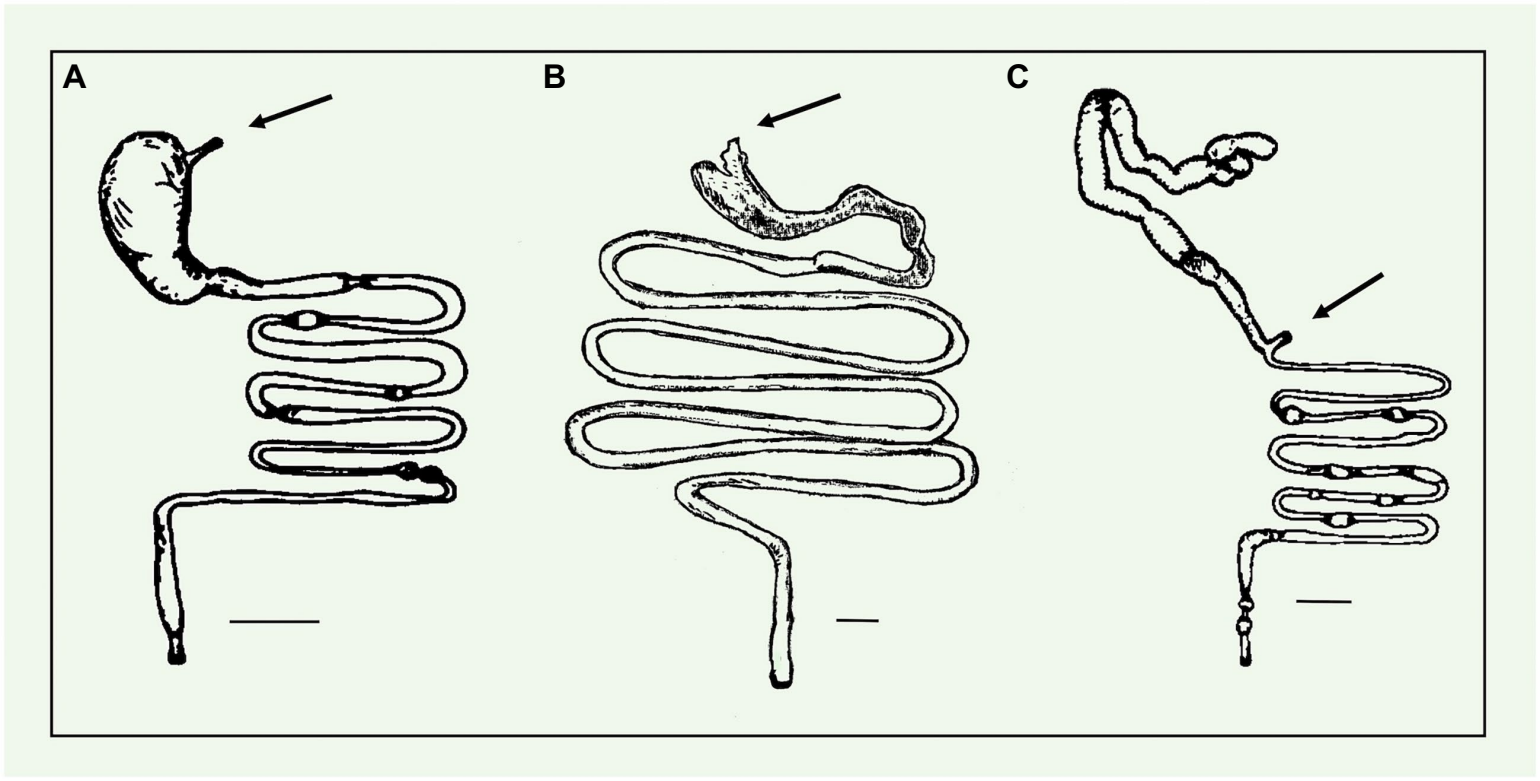
Gastrointestinal Tract. Schematic for gastrointestinal tracts of insectivorous **(A)**, frugivorous **(B)**, and sanguivorous **(C)** bats. Arrows point to the esophagus. Scale bar, 1 cm. **(A,C)** Modified from Stevens, (1980) and **(B)** modified from Tedman and Hall, (1985).

In bats, gut microbiota composition is associated to the extremely different feeding strategies that they exhibit, being either insectivory, frugivory, nectarivory, carnivory or sanguivory (Table 1; Dumont, 2007; Carrillo-Araujo et al., 2015; Ingala et al., 2019; Aizpurua et al., 2021). It has been demonstrated that several bacterial pathways, encoded in taxonomically different groups of microorganisms, are correlated with dietary specialization in bats, suggesting a role for bacteria in their ecological diversification (Ingala et al., 2021). The results obtained suggest that bats, across various feeding niches, may rely on their gut microbiota to fulfill essential metabolic roles that are related to the host dietary ecology. It has also been observed that the gut microbiota is affected by seasonal dietary (Xiao G. et al., 2019; Gong et al., 2021). As an example, in avivorous bats, the microbial community adapts to the change of feeding (from insects in summer to birds in spring and autumn), responding to increased energy demand for bird hunting and fat accumulation to hibernate and migrate (Gong et al., 2021). Furthermore, it was also observed that hibernation affects the intestinal microbiota of bats emphasizing a role of gut microbes in adapting hibernating animals to the extreme environment of fasting in winter (Xiao G. et al., 2019). The majority of bats are insectivorous (over 70% globally). Fruit bats, living in tropical areas, eat fruit and leaves, whereas some of them are specialized in a diet of pollen and nectar. In other cases, they eat birds, frogs, small animals, and even other bats. Three species are sanguivores and prey on large mammals like cows, sheep, and horses. This wide range of variation in diet is reflected in the structural features of their digestive system. Some of the major differences are seen in the gastrointestinal tracts (Figures 2A–C; Stevens, 1980; Tedman and Hall, 1985; Yani and Yuliyantika, 2019). As an example, the stomach of insectivores is unilocular and uncompartmentalized and its simplicity is probably due to the easily digestible proteins that constitute their diet (Aylward et al., 2019). The stomach of frugivores is bigger compared with insectivores, and it is relatively complex with compartmentalized areas which allow them to accumulate large quantities of food material (Tedman and Hall, 1985; Abumandour and Pérez, 2017). In the vampire species, the gastrointestinal system is different from the previous ones, and is specialized for a sanguineous diet. Haematophagous bats have a wide geographic range from Mexico to Central and South America, and they are the only mammals that feed exclusively of blood. Therefore, it can be hypothesized that they may carry a peculiar microbiota which can help them with blood digestion. They are represented by three species: *Desmodus rotundus* (common vampire bat), *Diphylla ecaudata* (hairy-legged vampire bat) and *Diaemus youngii* (white-winged vampire bat). Common vampire bat feeds preferentially on the blood from livestock, but also prey on wild animals and humans. *Diphylla ecaudata* and *D. youngii* feed on the blood from various birds, including poultry species. Indeed, unlike other mammals, in which the stomach separates the esophagus from the intestine, the vampire bats have a T-shape gastroesophageal-duodenal junction: one branch leads directly to the intestine, the other to the stomach (Figure 2C). The stomach forms a U shape blind-ending tube extremely long and thin (Rouk and Glass, 1970). Furthermore, the main functions of this organ are storage of large volume of blood and high water absorption (Mitchell and Tigner, 1970; Price et al., 2015).

The composition of the gut bacterial microbiota in phytophagous (both frugivorous and nectivorous) and insectivorous bats was examined in samples of fresh feces (Li et al., 2018). In both bat groups, although with significant percentual differences, the prevalent phylum is the Proteobacteria, with the major family of the *Enterobacteriaceae*, followed by Firmicutes and Tenericutes (Li et al., 2018). The *Enterobacteriaceae* family has been recently inserted into the Enterobacterales order. Enterobacterales is a new order which consists of seven families, including *Enterobacteriaceae* and *Yersiniaceae* (Adeolu et al., 2016). Unlike the other vertebrates, few members of the phylum of Bacteroidetes were found for both groups. The comparison between bats showed that the representative genera in phytophagous bats were *Weissella*, *Ureaplasma*, *Klebsiella, Enterobacter* and *Fructobacillus*, whereas the genera *Plesiomonas, Enterococcus, Lactobacillus*, and *Bacillus* were distinctive of insectivorous bats (Li et al., 2018).

In the guano of frugivorous *Rousettus leschenaulti*, the most prominent identified bacteria belong to the *Enterobacteriaceae* with genera *Enterobacter* and *Escherichia* followed by the genus *Enterococcus* of the *Enterococcaceae* family (Banskar et al., 2016).

*Yersinia enterocolitica* and *Yersinia pseudotuberculosis* are food and waterborne pathogens that cause enterocolitis in humans. They are widely present in the environment and are important zoonotic agents widespread in several animals. Both *Yersinia* species were isolated from bats (Table 1; Muhldorfer et al., 2010; Imnadze et al., 2020; Hahn et al., 2021). *Yersinia enterocolitica* was detected in the small intestine and spleen of dead bats (Muhldorfer et al., 2010; Imnadze et al., 2020). It is possible that the bacterium was the cause of death in animals, although this has not been yet elucidated (Imnadze et al., 2020). *Yersinia pseudotuberculosis* was detected in several organs, including lung, liver, and spleen (Muhldorfer et al., 2010; Hahn et al., 2021). Although the role of wild animals as reservoir hosts for *Yersinia* sp. is well known, in bats it has to be better clarified (Muhldorfer et al., 2010; Imnadze et al., 2020; Hahn et al., 2021).

*Campylobacter* is among the most common etiological agents of acute diarrhea in humans worldwide. Campylobacteriosis is an important foodborne zoonotic disease and is frequently related to handling and consumption of poultry meat (Igwaran and Okoh, 2019). Livestock animals, in particular poultry, are the major reservoir of *Campylobacter* species. *Campylobacter* was isolated and identified in rectal swab samples from the frugivorous *Rousettus amplexicaudatus*, resulting to be the second most predominant genus within the species *Campylobacter jejuni* and *Campylobacter coli* (Table 1; Hatta et al., 2016). Viable but non-culturable (VBNC) cells are living bacteria that do not either grow or divide on conventional laboratory media and do not develop into colonies (Li et al., 2014). The VBNC state is an adaptative strategy for extended survival of bacteria under stressful conditions. This state may be reversible, and it has been described for several human bacterial pathogens. *Campylobacter jejuni* remains in the environment, especially in water, in this VBNC state. It has been observed that the recovery of *C. jejuni* VBNC forms to culturability was obtained by passage through the mouse intestine (Baffone et al., 2006). Thus, *R. amplexicaudatus* may be a carrier of *C. jejuni* and it could be transmitted from bats to humans *via* water contaminated by their feces.

Considering the difficulty to obtain samples directly from wild individuals, a comparison between directly and indirectly collected samples was made to determine whether indirect sampling would produce results similar to direct sampling (Dietrich and Markotter, 2019). The results obtained showed that even if the sampling approach influenced the microbiota composition – i.e. cross-contamination in both methods or temporal sampling – niche specialization among excreta was well assessed by both methods (Dietrich and Markotter, 2019). Furthermore, significant differences in alpha-diversity microbial composition between small and large intestine and feces samples was observed in two insectivorous species supporting that fecal samples cannot be used as microbial inventories in other gut regions (Wu et al., 2019). Similar results were also obtained in another work suggesting that intestinal and fecal sampling methods are non-fungible (Ingala et al., 2018b). Although the two methods could give different information about the host, fecal samples are frequently used as surrogates for gut microbiota for several reasons (Ingala et al., 2018b; Tang et al., 2020). They are naturally collected, inexpensive, repeatable, and especially non-invasive. Moreover, it is not always possible to collect gut microbiota samples because several bat species are endangered, and they are protected by law in many countries.

*Anaplasma phagocytophilum* is an obligate intracellular Gram-negative bacterium and it is the etiological agent of human granulocytic anaplasmosis and tick-borne fever in domesticated animals (Jaarsma et al., 2019). The main vectors of bacterium are ticks of the *Ixodes ricinus* complex. Although numerous wildlife species can be infected, the consequence of *A. phagocytophilum* on their health is not known. The bacterial DNA of *A. phagocytophilum* was detected in the guano of 63 members of insectivorous *Rhinolophus hipposideros* (Table 1). Authors suggested that the high fecal DNA prevalence of the microorganism could be due to persistent infection but also to the consumption of insect preys carrying bacteria (Afonso and Goydadin, 2018). In this case, bat guano could be used as a bioindicator of the spread of *A. phagocytophilum* in the environment.

*Aeromonas hydrophila* was found in fecal samples of the sanguivorous *D. rotundus* (Table 1; Müller et al., 1980; Galicia et al., 2014). In humans, *Aeromonas* species cause gastrointestinal diseases as well as extraintestinal infections such as wound infections and septicemia. These microorganisms are ubiquitous in fresh and salty water. Like in other sanguivorous animals, *Aeromonas* appears to be necessary to digest blood meals producing proteolytic enzymes for decomposing its different components. It was observed that in young vampire bats, during the change from mothers’ milk to blood, coprophagy is common (Müller et al., 1980). These practices appear to be the natural way for inoculation of their intestine with the bacterium.

### Kidney

*Leptospira* – a genus of the *Leptospiraceae* family – is the etiological agent of leptospirosis that affects humans and animals. In humans, it causes a wide range of symptoms, and, in a second phase, it can lead to systemic severe illness up to multi-organ involvement. Leptospirosis outbreaks are associated with the presence of reservoir animals or accidental hosts that excrete the bacteria in their urine contaminating the environment. Leptospirosis is considered a public health problem in developing countries. Several studies have identified *Leptospira* spp. in bats (Table 1; Vashi et al., 2010; Bai et al., 2017; Dietrich et al., 2017; Ballados-González et al., 2018; Torres-Castro et al., 2020). The role of the bats as potential carriers of the *Leptospira* genus has been recognized (Vashi et al., 2010; Ballados-González et al., 2018). It has recently been observed that – on the basis of histopathological examination of bat renal tissue – the presence of inflammatory lesions were not significantly correlated with the presence of *Leptospira* in the kidney (Bevans et al., 2020). These results suggested that the animals were asymptomatically infected with the bacterium, supporting the hypothesis that bats’ kidneys may be reservoirs for zoonotic *Leptospira* (Bevans et al., 2020).

Recently, similar results were obtained analyzing bat kidney microbiota composition with metagenome analysis (Ramos-Nino et al., 2021). Several genera were identified, such as *Leptospira* and *Escherichia coli*. Furthermore, histopathological examination of the kidneys suggested that the bats analyzed were healthy and no lesions were observed. In accordance with previous results, the bats’ kidney can carry potential human pathogens (Ramos-Nino et al., 2021).

*Listeria monocytogenes* is an environmentally ubiquitous, intracellular bacterium that is pathogenic to humans and several animals. The disease is primarily transmitted by consumption of contaminated food. *Listeria monocytogenes* has been isolated from several wild animals, including bats (Höhne et al., 1975; Povolyaeva et al., 2020). To investigate the potential of *L. monocytogenes* to infect cells of bats, it was developed an *ex vivo* bat kidney epithelial cell line of *Pipistrellus nathusii* (Povolyaeva et al., 2020). The obtained data showed that *L. monocytogenes* invades and reproduces in bat kidney cells suggesting a similar mechanisms to those in humans (Povolyaeva et al., 2020).

### Blood

Several different hemiparasites have been observed in the blood of bats, including *Bartonella*, hemoplasmas, and trypanosomes protozoa. Protozoa will be discussed later.

*Hemotropic mycoplasmas* – of the genus *Mycoplasma* within the Tenericutes phylum and known as hemoplasmas – are pleomorphic, cell wall-less, uncultured, epicellular bacteria that parasitize erythrocytes in a wide range of vertebrate animals, including humans. Hemoplasmas can cause acute hemolytic anemia and several chronic diseases in hosts. The transmission route of bacteria includes blood-sucking arthropod vectors or transfer of infected blood. Hemoplasmas were identified in several hematophagous and non-hematophagous bat species (Table 1). The presence of hemoplasmas in the saliva of hematophagous bats suggests the possibility of direct transmission by biting, feeding on prey or social contact (Volokhov et al., 2017; Correia Dos Santos et al., 2020). In non-hematophagous bats, hematophagous arthropod vectors are considered the main form of transmission (Correia Dos Santos et al., 2020). It has been suggested that hemoplasma infection in bats is common and subclinical (Mascarelli et al., 2014; Millán et al., 2015). Furthermore, the strains are in close phylogenetic relationship with a human disease-causing microorganism, suggesting a role of bats as a natural reservoir of zoonotic pathogens (Mascarelli et al., 2014; Millán et al., 2015; Descloux et al., 2021).

*Bartonella* genus is a facultative intracellular Gram-negative, usually vector-borne bacteria, that colonize the endothelial cells and erythrocytes of several mammals, including humans and bats (Stuckey et al., 2017b). They are extremely heme-dependent, due to an inability to synthesize siderophore and to have a complete iron Fe^+3^ transport system (Biville and Liu, 2013). Members of the genus *Bartonella* have adapted to survive in a wide range of domestic and wild mammals without evidence of disease. *Bartonella* strains were described in both hematophagous and non-hematophagous bats and their arthropod ectoparasites (Table 1; Bai et al., 2017; Stuckey et al., 2017a,b; André et al., 2019; Corduneanu et al., 2021). The prevalence of strains was isolated from blood and other organ samples of *D. rotundus* and *D. ecaudata* probably due to their exclusive diet favorable to iron-deficient bacteria (Stuckey et al., 2017b; André et al., 2019). *Bartonella* has also been isolated in saliva samples suggesting that the bacterium could be transmitted between bats through behaviors that involve the transmission of saliva, such as biting and grooming (Dietrich et al., 2017).

## Fungi

The first report on the relationship between bats and fungi is from 1958 (Emmons, 1958). Afterwards, several fungi with both filamentous and yeast-like morphologies were recovered from bats (Botelho et al., 2012; Li et al., 2018; Ludwig et al., 2021).

It has been previously reported that the diversity of gut bacterial communities in bats are closely related to dietary changes. Fungi from bat gut are less studied. Recently, it has been demonstrated that the gut fungal communities are significantly affected by the dietary habits of the host, especially the gut mycobiota of phytophagous (frugivorous and nectarivores) bats as compared to insectivores (Li et al., 2018). This is probably due to the preference of frugivores to eat mature fruits. Indeed, considering that fungi are involved in fermentative processes, the fermented fruits may be the primary source of the fungi. Fungi were detected in all the fecal samples tested, and the prevalent phyla were Ascomycota and Basidiomycota. The number of fungi in the feces of phytophagous bats was relatively higher than in insectivores. Most of the fungi are foodborne and are also pathogens of humans and other animals.

*Pseudogymnoascus destructans* is the etiological agent of white-nose syndrome (WNS) responsible for the death of millions of bats in North America (Hoyt et al., 2021). It is a psychrophilic fungus that infects the skin of bats during the winter season while they are in hibernation. The fungus can invade the living tissue of the animal causing the characteristic severe skin lesions. Molecular studies indicated that *P. destructans* is native of Eurasia, suggesting a recent introduction of this fungus in North America (Fritze et al., 2021). In Europe, *P. destructans* is not associated with mass mortality indicating that European bats may have evolved an effective immune defense (Fritze et al., 2021). The disastrous population reduction in North America bat communities could persist for many decades, also considering the slow growth rate of these animals. Therefore, it is necessary to focus on research to prevent spread and mitigate impacts. At present, methods to prevent WNS are limited (Hoyt et al., 2021). Bat skin microbiota is also strongly influenced by complex and interacting factors and the skin microbiota can influence the growth of other microorganisms (Hoyt et al., 2015; Hamm et al., 2017; Lemieux-Labonté et al., 2017; Li Z. et al., 2021). As previously reported, bacteria present on the skin may play a key role in counteracting the progression and outcome of the disease (León et al., 2009; Hoyt et al., 2015, 2019; Cheng et al., 2017; Hamm et al., 2017; Grisnik et al., 2020; Li A. et al., 2021; Li Z. et al., 2021).

Bat skin bacteria could be used as biocontrol agents to influence the disease outcomes and thereby protect bats exposed to the fungus (Cheng et al., 2017; Hoyt et al., 2019). Hoyt et al. have tested the efficacy of *Pseudomonas fluorescens* as a probiotic bacterium to reduce the effects of WNS on caged and free-flying *Myotis lucifugus* (Hoyt et al., 2019). The results suggested that *P. fluorescens* could be a useful tool to reduce the impacts of WNS. Testing the efficacy of *P. fluorescens* on WNS on other species would be important considering that there are species – such as *Myotis septentrionalis* – at risk of extinction due to *P. destructans*.

It has also been reported that some yeast strains isolated from bat wings in western North America are strongly associated with resistance to WNS (Vanderwolf et al., 2021b). These microorganisms were tested for *P. destructans*-antagonistic properties by spore germination and growth inhibition/competition assays, and their ability to inhibit *P. destructans in vitro* was confirmed. Similar results were obtained with yeasts isolated from bat wings in eastern North America (Vanderwolf et al., 2021a).

*Histoplasma capsulatum* is a dimorphic pathogenic fungus of mammals, which causes pulmonary and systemic infections in humans, and it is acquired *via* inhalation of the fungal spores. *Histoplasma capsulatum* is commonly found in soil associated with great amounts of birds’ droppings or to bats guano. Histoplasmosis is an endemic mycosis and it is a common opportunistic infection among patients with advanced AIDS or weakened immune system for other reasons (Myint et al., 2020). Association of bats with histoplasmosis dates back to the late 1950s (Emmons, 1958). Indeed, bats are considered as the main reservoirs and dispersers of this fungus in the environment (Taylor et al., 2012). Moreover, it was observed that subjects occupationally exposed to bat sites, have high risk of infection and can develop severe clinical forms of histoplasmosis (Santos et al., 2013). *Histoplasma capsulatum* has been identified and isolated in several bat species (Dias et al., 2011; González-González et al., 2014; da Paz et al., 2018; Vite-Garín et al., 2021). A co-infection with *H. capsulatum* and *Pneumocystis* spp. was also observed (Table 2; González-González et al., 2014). *Histoplasma capsulatum* and *Pneumocystis* share several features, such as low pathogenicity in healthy hosts and severe disease in immunocompromised hosts; they use the respiratory portal of entry and have the ability to disseminate from the lungs to other organs (González-González et al., 2014). *Pneumocystis* is a genus of closely related unicellular fungi of low virulence found in the lungs of humans and several mammals.

**Table 2:** Taxonomic distribution of commonly widespread eukaryote in bats.

Bat species	Status/Diet	Samples	Detection and identification approach^a^	Taxonomic distribution at level of		Ref.
				Phyla	Genera/Species	
*Fungi*						
*Rousettus leschenaultia, Cynopterus sphinx*	Free-living/F	Feces	Molecular	Ascomycota	*Candida albicans*, *Geotrichum candidum, Hanseniaspora* sp.	Li et al. (2018)
*Molossus molossus, Nyctinomops macrotis, Tadarida brasiliensis, Molossus rufus, Eumops glaucinus*	Free-living/F	Spleens and livers	Traditional	Ascomycota	*Histoplasma capsulatum*	Dias et al. (2011)
*Artibeus hirsutus* *Glossophaga soricina* *Natalus stramineus* *Tadarida brasiliensis* *Myotis californicus*	Free-living/F Free-living/P,I Free-living/I Free-living/I Free-living/I	Lung	Molecular	Ascomycota	*H. capsulatum* *Pneumocystis* sp.	González-González et al. (2014)
*Sturnira lilium*	Free-living/F	Feces	Traditional	Ascomycota	*C. parapsilosis, C. krusei,* *C. parapsilosis*	Botelho et al. (2012)
*Artibeus fimbriatus*	Free-living/F	Feces	Traditional	Ascomycota	*C. parapsilosis*	Botelho et al. (2012)
*A. lituratus*	Free-living/F	Feces	Traditional	Ascomycota	*C. pelliculosa, C. lusitaniae,* *C. guilliermondii*	Botelho et al. (2012)
*Molossus molossus*	Free-living/I	Intestine	Traditional	Ascomycota Basidiomycota	*Penicillium* sp., *Fusarium* sp., *Aspergillus* sp., *C. albicans*	Ludwig et al. (2021)
*Eonycteris spelaea*	Free-living/N	Feces	Molecular	Ascomycota	*C. albicans*, *G. candidum*	Li et al. (2018)
*Myotis ricketti, Hipposideros larvatus, Tylonycteris pachypus, Pipistrellus abramus, Scotophilus heathi, Hipposideros armiger*	Free-living/I	Feces	Molecular	Ascomycota	*Cladosporium* spp., *Blastobotrys terrestris*	Li et al. (2018)
*Protozoa*						
*Artibeus cinereus*	Free-living/F	Blood	Molecular	Euglenozoa	*Leishmania amazonensis*	Vieira et al. (2022)
*Molossus molossus* *Desmodus rotundus*	Free-living/I Free-living/S	Heart and pectoral muscle	Bioassays	Apicomplexa	*Toxoplasma gondii*	Cabral et al. (2013)
*Tadarida brasiliensis*	Free-living/I	Vascular wing membrane	Molecular	Euglenozoa	*Trypanosoma cruzi*	Nichols et al. (2019)
*Desmodus rotundus* *Histiotus montanus*	Free-living/S Free-living/I	Plagiopatagium Feces, perianal	Molecular	Euglenozoa	*Trypanosoma cruzi*	Quiroga et al. (2022)
*Desmodus rotundus* *Diphylla ecaudata* *Glossophaga soricina* *Carollia perspicillata*	Free-living/S Free-living/S Free-living/N Free-living/F	Saliva	Molecular	Euglenozoa	*Trypanosoma cruzi*	Bergner et al. (2021)
*Myotis ciliolabrum* *Pipistrellus pipistrellus*	Free-living/I	Feces	Molecular	Apicomplexa	*Cryptosporidium parvum*	Kváč et al. (2015)
*Pteropus conspicillatus*	Captive/F	Feces	Molecular	Apicomplexa	*Cryptosporidium hominis*	Schiller et al. (2016)

a Traditional identification methods are based on the isolation in culture media of microorganisms followed by biochemical tests. Molecular techniques rely on the analysis of genomic markers corresponding to nucleic acid sequences directly from the clinical specimen without need for prior culture. When present, both methods are used in succession: Traditional to isolate bacteria of interest and molecular for identification. F, Frugivores; N, Nectarivores; I, Insectivores; S, Sanguivores; P, Phytophage.

The *Candida* genus comprises a heterogeneous group of opportunistic yeast Ascomycota in the normal microbiota of the mucosa oral cavity, the gastrointestinal tract and vagina in healthy people (Sardi et al., 2013). In immunocompromised conditions, they are responsible for several types of human disease from local mucocutaneous overgrowth to invasive systemic infections. Several studies on *Candida* spp. are also reported from bats (Table 2; Botelho et al., 2012; Brilhante et al., 2016; Li et al., 2018). As an example, five species of the genus *Candida* were identified in the feces of seven urban frugivorous bats, in different areas of Londrina city in Brazil (Botelho et al., 2012). Furthermore, several fungal genera were isolated in an urban forest fragment in Sinop city in Brazil (Ludwig et al., 2021). In different bats – prevalently insectivores – both filamentous and yeast fungi such as *Aspergillus* spp., *Fusarium* spp. *Cryptococcus* spp. and *Candida* spp. which may cause opportunistic infections in humans were isolated (Table 2; Ludwig et al., 2021). The presence of bats in an urban area suggests that the environment can be contaminated with their feces and that inhabitants are exposed daily to these microorganisms (Botelho et al., 2012; Ludwig et al., 2021).

## Archaea

Unlike in humans where archaea are naturally occurring components of the human gut microbiota (Gaci et al., 2014), there is no evidence that these microorganisms are present in bats. In a previous paper, diverse archaeal communities in bat guano were identified and sequenced, but whether these microorganisms are also present in the bats gut is presently unknown (Chronáková et al., 2009). However, the use of appropriate archaea-specific primers could lead to the identification of these microorganisms which would otherwise be difficult (Hathaway et al., 2021).

## Protozoa

Protozoa are eukaryotic unicellular microorganisms found widespread in several habitats. Protozoan infections range from asymptomatic to severe diseases, depending on the parasite and the resistance of the host. In humans, the major protozoan diseases are malaria, leishmaniasis, toxoplasmosis, trypanosomiasis, and cryptosporidiosis. Bats are also hosts of several protozoan species for some of which they are considered reservoirs. To date, no correlation between humans and bats on malaria has been observed. Infections in humans are caused by six species of the *Plasmodium* genus of the Haemosporidia order (Sutherland and Polley, 2017). However, several other malaria-related haemosporidian parasites are present in wildlife populations, including bats (Perkins and Schaer, 2016).

*Toxoplasma gondii* is a zoonotic, obligate intracellular protozoan parasite that causes the disease toxoplasmosis and it has a worldwide distribution (Attias et al., 2020). The microorganism infects several species of warm-blooded animals, including humans. Humans usually become infected through the ingestion of protozoan cysts in contaminated meat. *Toxoplasma gondii* may be also transmitted vertically passing to the fetus *via* the placenta and it is associated with miscarriage and severe birth defects. Studies about the role of bats in toxoplasmosis are limited. Information about the presence of *T. gondii* in bats were obtained through detection of antibodies and of the DNA of the microorganism (Yang et al., 2021). Viable parasite was isolated – through inoculation in mice – from heart and pectoral tissues of an insectivorous and a hematophagous bat (Table 2; Cabral et al., 2013). Molecular techniques were used to genotype the samples. The two genotypes isolated in bats had already been described previously in other wild and domestic animals suggesting the circulation of the strains in some geographic areas.

Leishmaniasis is a tropical disease typical of numerous mammals, including humans (Gradoni, 2018). At least 20 recognized *Leishmania* species are pathogenic to humans, and they are primarily transmitted by the bite of an insect vector of the genera *Phlebotomus* and *Lutzomyia*. In humans, visceral and cutaneous leishmaniasis are the major clinical forms prevalent worldwide (Gradoni, 2018). Bats were identified as potential reservoirs of several *Leishmania* species such as *L. braziliensis, L. mexicana, L. infantum*, and *L. amazonensis* (Maia et al., 2018; Vieira et al., 2022). In several studies, the protozoan was detected in liver, spleen, and skin. Recently, *Leishmania* was also detected in blood samples (Vieira et al., 2022). It has been observed that *Leishmania* infection rates were higher in frugivorous bats (Table 2).

*Trypanosoma* genus comprises hematophagous protozoans distributed worldwide than can infect several mammals. In humans, trypanosomiasis are chronic diseases that are endemic in parts of Africa and South America. African trypanosomiasis, caused by the subspecies *Trypanosoma brucei rhodesiense* and *Trypanosoma brucei gambiense*, is transmitted by the hematophagous tsetse fly and causes meningoencephalitis in which somnolence is a prominent characteristic. In bats, the subspecies *Trypanosoma brucei brucei* has been detected, a parasite primarily of cattle and occasionally of other animals, where it causes similar neurological disorders (Cai et al., 2019). American trypanosomiasis – or Chagas disease – is caused by *Trypanosoma cruzi* that is mainly transmitted to mammals by infected feces of blood-sucking bugs. As insects bite the hosts they defecate, and protozoans enter through the skin wound. The disease is associated with a frequently asymptomatic chronic phase, which can last for decades, and a highly mortal acute phase with cardiac, neurological, and gastrointestinal complications. The protozoan is also transmitted by eating infected triatomine insects or their feces.

Several species of bats have been reported as hosts of *T. cruzi* (Hodo et al., 2016; Nichols et al., 2019; Bergner et al., 2021; Torres-Castro et al., 2021; Quiroga et al., 2022). For instance the presence of *T. cruzi* in the saliva of four Neotropical bat species in northern Peru was reported (Table 2; Bergner et al., 2021). Two of them are hematophagous bat species, and given the regional significance of Chagas disease, authors underlined the need for further research into the potential risk of zoonotic transmission directly from bat bites. *T. cruzi* was also detected in a migratory bat species in Oklahoma (Nichols et al., 2019; Table 2). Endemicity of the protozoan in Oklahoma is probably due to the annual migration of these bats from Mexico. Several other species of *Trypanosoma* genus have been detected in bats and they are exclusive of bats and other animals (Sato and Mafie, 2022).

*Cryptosporidium* spp. and *Giardia duodenalis* are common etiological agents of diarrheal diseases in humans and animals worldwide (Dixon, 2021; Ryan et al., 2021). Transmission of both parasites occurs by the fecal-oral route through direct contact with infected humans or animals, or indirectly (*via* water or food). Numerous *Cryptosporidium* species and genotypes that have been identified are able to infect humans. In humans, *Cryptosporidium parvum* and *Cryptosporidium hominis* are the most relevant species (Ryan et al., 2021). Several *Cryptosporidium* species have been detected in bats (Kváč et al., 2015; Schiller et al., 2016; Li et al., 2019; Adhikari et al., 2020). In particular, the human pathogenic *C. parvum* was identified in two insectivores bats from United States and Czech Republic areas (Table 2; Kváč et al., 2015). Furthermore, the presence of human specific *C. hominis* in captive flying foxes in Australia was reported (Table 2; Schiller et al., 2016). Although the role of bats in the transmission of *Cryptosporidium* spp. to humans remains to be clarified, these results highlight the potential for transmission of these microorganisms. Finally, although giardiasis is a very common disease in humans as well as in a large number of mammals, little is known about the presence of *Giardia* species in bats (Adhikari et al., 2020).

**Figure 3:**
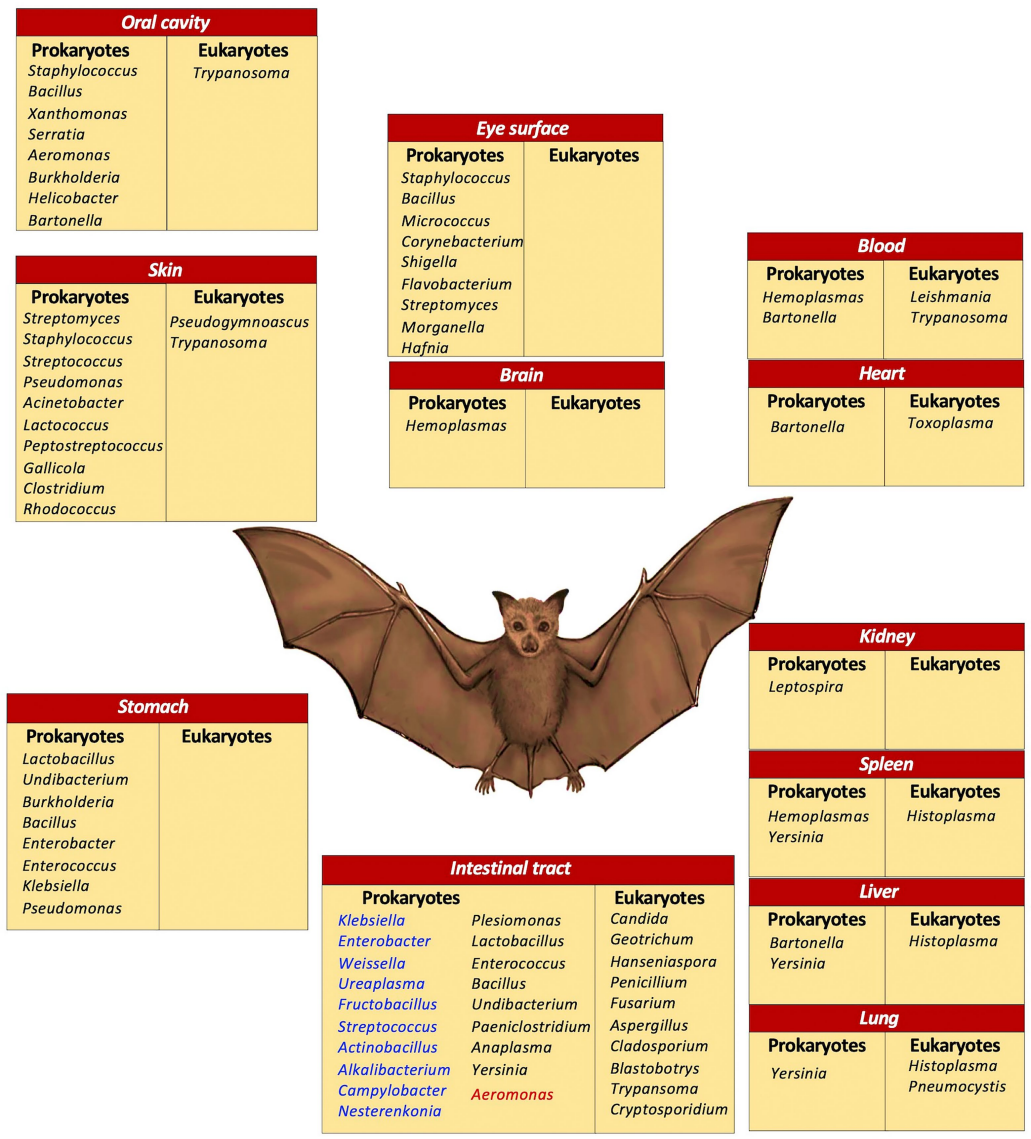
Microbial diversity in bats. A compilation of microorganisms at genera level in various body sites. Intestinal tract: blue, frugivores and nectivores; black, insectivores; red, sanguivores.

Figure 3 illustrates the microorganisms at genera level, according to the various body sites where they were found, discussed in this work.

## Antimicrobial resistance in bats

**Figure 4:**
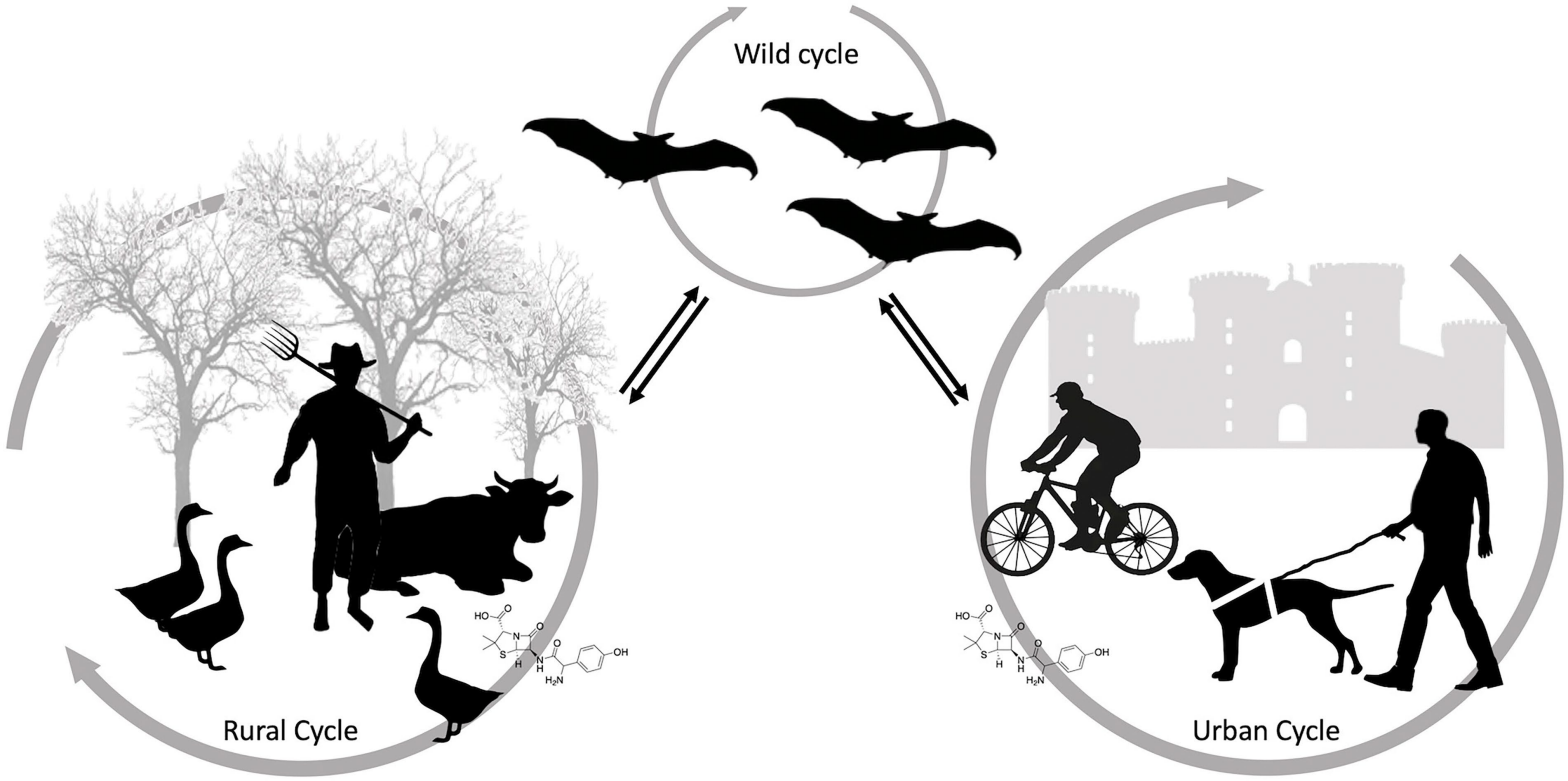
Schematic representation of antimicrobial resistance transmission pathways between anthropogenic and natural ecosystems.

Antimicrobial resistance (AMR) is a major and increasing global healthcare problem. The misuse or excessive use of many antibacterial drugs in both healthcare and agriculture are considered to be the main drivers of antimicrobial resistance (Dadgostar, 2019). Over time, antimicrobial drugs become ineffective, and infections become progressively difficult or impossible to treat (e.g., Extensively Drug-Resistant Tuberculosis). AMR may be also considered as an ecological problem and is characterized by complex interactions between humans, domestic and wild animals and the environment (McEwen and Collignon, 2018). AMR is one of the most relevant areas of work of the “One Health” approach (Figure 4). One Health is a healthcare approach based on the integration of various fields of science. It is based on the recognition that human health, domestic animals and wildlife health, and environmental health are indissolubly linked (McEwen and Collignon, 2018). Although wildlife species can naturally harbor antibiotic resistant bacteria, they are not normally exposed to antibacterial drugs and can acquire resistant bacteria through contact with humans, livestock, domestic animals and the environment (Kozak et al., 2009). Selective pressure exerted by humans may increase the potential for wild animals to carry emerging resistant bacteria and support their dissemination (Dolejska, 2020). Indeed, it has been observed that animals living in close contact with humans are more likely to carry resistant bacteria than those in areas with limited or absent anthropogenic activities.

AMR was also observed in synanthropic bats, which can contribute to the spreading of resistant bacteria in the environment as well as to transmit them to humans. Bats have adapted to living in close proximity to human connected with their life cycle and habitat. Multi-resistant bacterial strains were isolated and identified in bats (García et al., 2020;Nowakiewicz et al., 2020; McDougall et al., 2021; Obodoechi et al., 2021; Benavides et al., 2022). Although different bacteria are important in terms of antimicrobial resistance in humans, extended-spectrum beta-lactamases (ESBL) producing Gram-negative bacteria like *E. coli* are considered key indicator pathogens to study the evolution of multi-resistant bacteria in the environment and wildlife (Radhouani et al., 2014). ESBL-producing *E. coli* isolates from bats are reported worldwide (Nowakiewicz et al., 2020; McDougall et al., 2021; Obodoechi et al., 2021; Benavides et al., 2022). ESBLs are enzymes that confer resistance to most beta-lactam antibiotics, including penicillins and cephalosporins. Infections with ESBL-producing microorganisms have been associated with poor outcomes. Genes encoding resistance against various antibacterial agents, such as aminoglycosides and trimethoprim, were also detected (McDougall et al., 2019). The relationship between ARM and the selective pressure of anthropogenic activities is confirmed by the observation that in bats living in uncontaminated areas a lower number of resistant bacteria was found (Cláudio et al., 2018).

## Bat host–microbe interactions: Health or disease

Immunological tolerance is the ability of the immune system to coexist with potentially antigenic self-molecules, cells, and tissues (Abul et al., 2019). The immune system is also tolerant against products of commensal microbes that live in symbiosis with the host. In humans, the gut microbiota aids in the digestion and absorption of foods and prevents overgrowth of potential pathogens. The immune system can recognize these microorganisms and does not react against them. Also, intestinal microbiota is an important player in health and disease (Fan and Pedersen, 2021). In bats, immunological tolerance has been observed against viral infections (Guito et al., 2021; Irving et al., 2021; Sia et al., 2022). In this case, the immune response is not directed primarily against the pathogen through an inflammatory response, but instead at limiting host tissue damage caused by pathogen and activating tissue repair mechanisms. Recent studies on the bat immune system have highlighted a possible link between the evolution of flight in bats and viral persistence (Subudhi et al., 2019). Furthermore, it has been suggested that the bat gut microbiota could contribute to immunological tolerance to viruses (Luo et al., 2021, 19; Popov et al., 2021). However, the exact mechanisms have not been yet revealed.

The host-microorganism interaction is a dynamic process in which each one acts to maximize its survival. The host-associated microorganisms are influenced by various surrounding conditions, including host conditions, abiotic factors, different food habits and interactions between them (Figueiredo and Kramer, 2020). The composition and diversity of microbial species will vary according to the characteristics of the host and the environment. While most of interactions between hosts and microorganisms do not result in disease, the relationships can have a negative effect – i.e., involving a molecular and cellular response – on host performance (Figueiredo and Kramer, 2020). In humans, gut microbiota is involved in several metabolic diseases (Fan and Pedersen, 2021). Like in humans and other animals, gut microbiota in bats plays an important role in digestion, immunity, and health. Recently, it has been observed that bats with specialized diets may partially rely on their gut microbiota to satisfy or increase critical nutritional pathways, including essential amino acid synthesis, fatty acid biosynthesis, and the generation of cofactors and vitamins essential for proper diet (Ingala et al., 2021).

## Conclusion

As highlighted in the previous paragraphs, bats are unique among mammals in many ways, if we consider their distinguishing features such as the ability to fly, the different feeding strategies and their world-wide distribution. Here we have focused on the bats microbiota and tried to offer a comprehensive view of the current knowledge about the microorganisms that live together with bats, and their distribution in bats tissues, an issue that we feel is currently understudied especially if we compare it with the wide literature available on bats’ viruses. This gap may be the result of the fact that many of the microorganisms that were found in the different bats body districts are uncultivable *in vitro*, despite the newest progresses.

However, further studies on bat microbiota are really in demand in view of their epidemiological significance. Indeed, we have highlighted that the more deeply we go into the knowledge of microorganism that populate bats organs, the more pathogens to humans and livestock we identify. Furthermore, given the anthropocentric modifications that we are continuously pursuing to the environment, our promiscuity with the different bats species is ever increasing, and this makes us every day more exposed to the above-mentioned pathogens, with the consequent local or global health problems arising from transmissible infections.

Under this light we anticipate that particular attention will have to be devoted to the identification of AMR in bats. Indeed, bats may be among the main species contributing to the spreading of resistant bacteria in the environment and, consequently to humans, both through the food chain or direct contact. A deeper knowledge of bats microbiota, especially of those that are in closer contact with humans, may therefore help in preventing difficult-to-treat bacterial infections.

## Author contributions

NA and LF wrote the manuscript. MM carried out the bibliography and performed the tables. VDL critically read the manuscript before submission. All authors have read and approved the final version of the manuscript.

## Funding

This work was partly supported by grants from the University of Chieti-Pescara “G. d’Annunzio” (to LF and NA).

## Conflict of interest

The authors declare that the research was conducted in the absence of any commercial or financial relationships that could be construed as a potential conflict of interest.

## Publisher’s note

All claims expressed in this article are solely those of the authors and do not necessarily represent those of their affiliated organizations, or those of the publisher, the editors and the reviewers. Any product that may be evaluated in this article, or claim that may be made by its manufacturer, is not guaranteed or endorsed by the publisher.
